# Tablet-based Augmented reality and 3D printed templates in fully guided Microtia Reconstruction: a clinical workflow

**DOI:** 10.1186/s41205-024-00213-2

**Published:** 2024-05-31

**Authors:** Alberto Díez-Montiel, Alicia Pose-Díez-de-la-Lastra, Alba González-Álvarez, José I. Salmerón, Javier Pascau, Santiago Ochandiano

**Affiliations:** 1 Instituto de Investigación Sanitaria Gregorio Marañón, Madrid, 28007 Spain; 2 Servicio de Cirugía Oral y Maxilofacial, Hospital General Universitario Gregorio Marañón, Madrid, 28007 Spain; 3 Departamento de Bioingeniería, Universidad Carlos III de Madrid, Leganés, 28911 Spain

**Keywords:** Reconstructive surgery, microtia correction, 3D printing, Augmented reality, Intraoperative guidance.

## Abstract

**Background:**

Microtia is a congenital malformation of the auricle that affects approximately 4 of every 10,000 live newborns. Radiographic film paper is traditionally employed to bidimensionally trace the structures of the contralateral healthy ear in a quasi-artistic manner. Anatomical points provide linear and angular measurements. However, this technique proves time-consuming, subjectivity-rich, and greatly dependent on surgeon expertise. Hence, it’s susceptible to shape errors and misplacement.

**Methods:**

We present an innovative clinical workflow that combines 3D printing and augmented reality (AR) to increase objectivity and reproducibility of these procedures. Specifically, we introduce patient-specific 3D cutting templates and remodeling molds to carve and construct the cartilaginous framework that will conform the new ear. Moreover, we developed an in-house AR application compatible with any commercial Android tablet. It precisely guides the positioning of the new ear during surgery, ensuring symmetrical alignment with the healthy one and avoiding time-consuming intraoperative linear or angular measurements. Our solution was evaluated in one case, first with controlled experiments in a simulation scenario and finally during surgery.

**Results:**

Overall, the ears placed in the simulation scenario had a mean absolute deviation of 2.2 ± 1.7 mm with respect to the reference plan. During the surgical intervention, the reconstructed ear was 3.1 mm longer and 1.3 mm wider with respect to the ideal plan and had a positioning error of 2.7 ± 2.4 mm relative to the contralateral side. Note that in this case, additional morphometric variations were induced from inflammation and other issues intended to be addressed in a subsequent stage of surgery, which are independent of our proposed solution.

**Conclusions:**

In this work we propose an innovative workflow that combines 3D printing and AR to improve ear reconstruction and positioning in microtia correction procedures. Our implementation in the surgical workflow showed good accuracy, empowering surgeons to attain consistent and objective outcomes.

## Background

Augmented reality (AR) has emerged as a groundbreaking technology revolutionizing the visualization of complex three-dimensional structures seamlessly integrated into the real environment [1, 2]. In the realm of surgical guidance, this tool is on-the-edge [3, 4], as it has the potential to shorten the learning curve of these procedures and ultimately increase overall efficiency and patient safety [5]. Its successful implementation spans across various specialties, including neuronavigation [6–9], dental surgery [10], orthopedics [11–13], oncology [14, 15], and neurology [16]. Along with all these works, AR has already proven its efficacy in highlighting anatomical structures in patients, displaying cutting planes and providing guidance for the precise placement of surgical tools [17, 18].

One great complement to AR is 3D printing. It enables the production of cost-effective, personalized components that significantly enhance clinical procedures. Extensive research has shown that the potential errors introduced by various materials and printers are sufficiently low to warrant reliance on this technology [19]. As such, 3D printing already encompasses a wide range of applications in the medical field, such as optimizing the understanding of complex patient conditions [20], facilitating preoperative planning [21], creating custom implants [22], and improving communication with patients [23]. When combined with AR, 3D printing serves as a powerful bridge between the real and virtual worlds. In particular, our group has developed innovative solutions that integrate these two technologies to present patient-specific information during surgical interventions [17, 18, 24]. Our approach involves 3D-printed surgical guides that uniquely fit on a particular location of the patient’s bone. In the surgical room, an AR reference marker can be easily attached to these guides. Knowing the relative coordinates between the AR reference marker, the surgical guide, and the patient, we can seamlessly augment patient’s specific information *on site*.

The accuracy of AR projections in relation to real anatomical structures has been previously evaluated in simulation scenarios [24]. However, the precision of this AR approach for surgical guidance has not yet been determined during real clinical interventions. In this work, we developed an in-house AR application compatible with any commercial Android device based on the one presented in [24]. It was specifically tailored to provide guidance during microtia correction procedures, which treat patients with ear malformations. A more detailed explanation of the clinical context of this condition and its conventional treatment is presented in 1.1.

Our solution assists throughout the entire surgical workflow. First, we introduce patient-specific 3D printed cutting templates and remodeling molds to objectively carve and construct the new ear. Second, our AR application displays the optimal location of the new ear on the patient’s head to guide its placement during surgery. We propose our AR solution in the form of a tablet application because this format offers a virtual experience that is both ubiquitous and affordable. Furthermore, it facilitates a shared view among all surgeons in the operating room, fostering collaborative decision-making during the procedure. The system was first evaluated on a simulation scenario and then employed to guide an actual surgery. Supplementary material referred to in this work is available at https://zenodo.org/records/10958624. All 3D models employed to analyze the results are contained within the folder SuppContent_3Dmodels.

### Microtia correction procedures

Microtia is a congenital malformation of the auricle [25]. It can range from a diminutive ear with intact anatomical structure to a total absence of the ear, referred to as anotia. Its incidence varies among different series, but it is estimated to affect approximately 4 of every 10,000 live newborns. In 90% of cases, microtia is unilateral, with the right side affected in 60% of patients. Additionally, it occurs more frequently in males, accounting for around 65% of the cases. While the exact etiology of microtia remains unknown, it is believed to have multifactorial origins involving both environmental and genetic factors.

The rib graft technique, pioneered by Dr. Radford Tanzer in 1959 [26], stands as the gold standard for ear reconstruction. He introduced the utilization of autologous rib cartilage in multiple stages to reconstruct the ear. In the 1990s, Dr. Françoise Firmin further refined this technique, establishing reproducible outcomes for reconstructive surgeons through a two-step process [27]. First, a cartilaginous framework is meticulously crafted to form the new ear. Its shape and proportions are traditionally obtained from the contralateral healthy ear by tracing each structure using radiographic film paper. In the subsequent step, the new ear is positioned as symmetrically as possible using reference measurements from the external canthus of the eye to the root of the helix and from the labial commissure or nasal wing to the earlobe. For the angulation of the new ear, the references utilized are the long axis of the ear and the tangential line of the nasal dorsum. However, this technique is highly subjective and heavily reliant on the surgeon’s experience, making it susceptible to shape errors and mispositioning.

Depending on the specific type of microtia, different structures must be reconstructed. In cases where the ear lacks identifiable anatomical landmarks, the cartilaginous framework typically comprises a minimum of four pieces: the base, helix, antihelix, and tragus-antitragus. These components are carefully crafted and then housed within a cutaneous pocket created on the patient’s head. The entire procedure is typically conducted in two subsequent surgeries, spaced approximately six months apart. During the first surgery, the cartilaginous framework is constructed and placed within the cutaneous pocket. The retroauricular groove is not reconstructed, and hence the ear is entirely connected to the head. In the second surgery, the retroauricular groove is reconstructed to project the ear and achieve a natural appearance [28].

## Methods

The following subsections delve into a comprehensive explanation of the methodology employed throughout this work. Specifically, 2.1 presents the patient participating in this study. Then, 2.2 elaborates on the design of the preoperative plan. 2.3 explains the 3D printing techniques employed along with the adherence to sterilization requirements mandated by the operating room’s constraints. The development and functionality of the AR application is detailed in 2.4. Finally, 2.5 presents the technical and functional evaluation of the work, as well as the steps followed during the surgical procedure.

### Clinical data

**Fig. 1:**
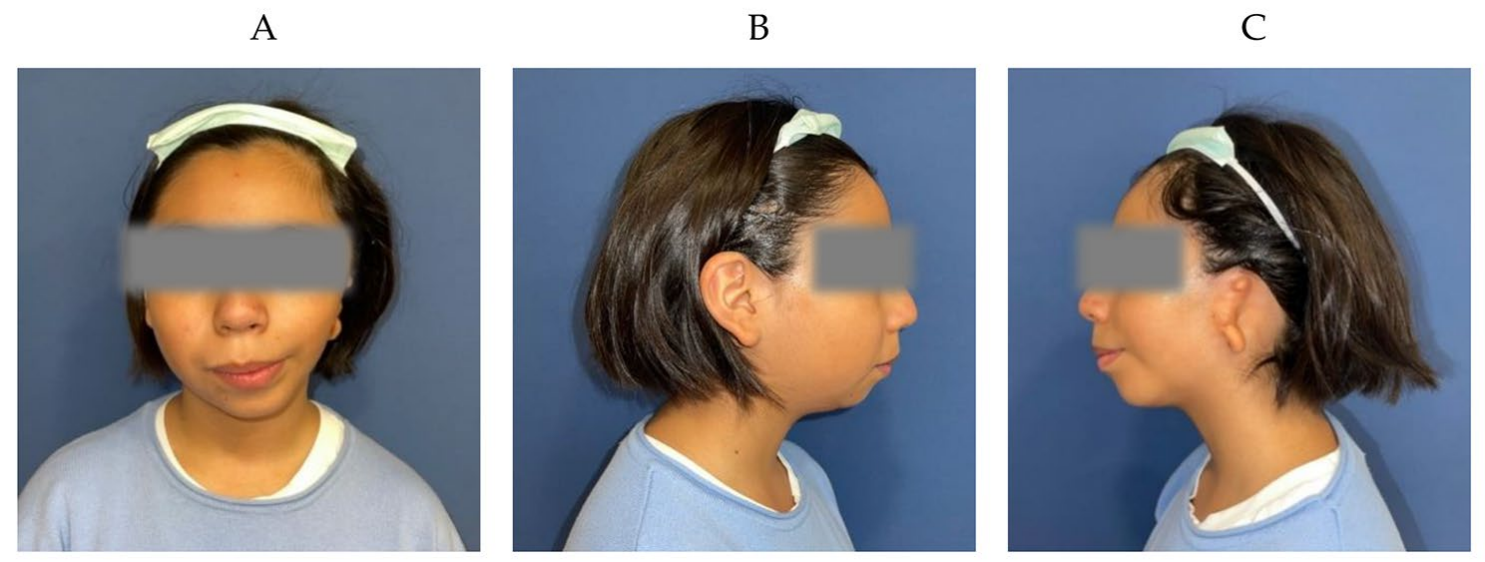
Patient presenting left hemifacial microsomia with a lobular type microtia. (**A**) Frontal view. (**B**) Right side view. (**C**) Left side view showing microtia

We present the case of an 11-year-old girl with left hemifacial microsomia treated at Hospital General Universitario Gregorio Marañón (HGUGM) in Madrid, Spain. According to Nagata´s classification, the patient presents a lobular type microtia (Fig. 1) [29]. Due to the absence of usable cartilaginous remnants, the treatment assigned to the patient involved a complete reconstruction of the cartilaginous framework, including base, helix, antihelix, and tragus-antitragus complex.

### Surgical plan design

Two preoperative CT scans of the patient’s head and thorax were acquired on a Philips Mx8000 CT scanner with a slice thickness of 0.5 mm. The resulting DICOM files were exported to 3D Slicer software to generate virtual 3D biomodels. The ideal shape and location of the reconstructed ear was obtained from the first scan by mirroring the healthy ear with respect to the sagittal axis of the head (SuppVideo1_SurgicalPlanDesign). The resulting structure is referred to as the specular ear. We also designed a custom dental splint using *Blue Sky Plan* software (Blue Sky Bio Company) that securely fits over the patient’s upper teeth. It played a crucial role in facilitating automatic registration within the AR application. Lastly, we created a phantom comprising part of the patient’s skull from the head’s CT scan to validate the system in the simulation scenario.

**Fig. 2:**
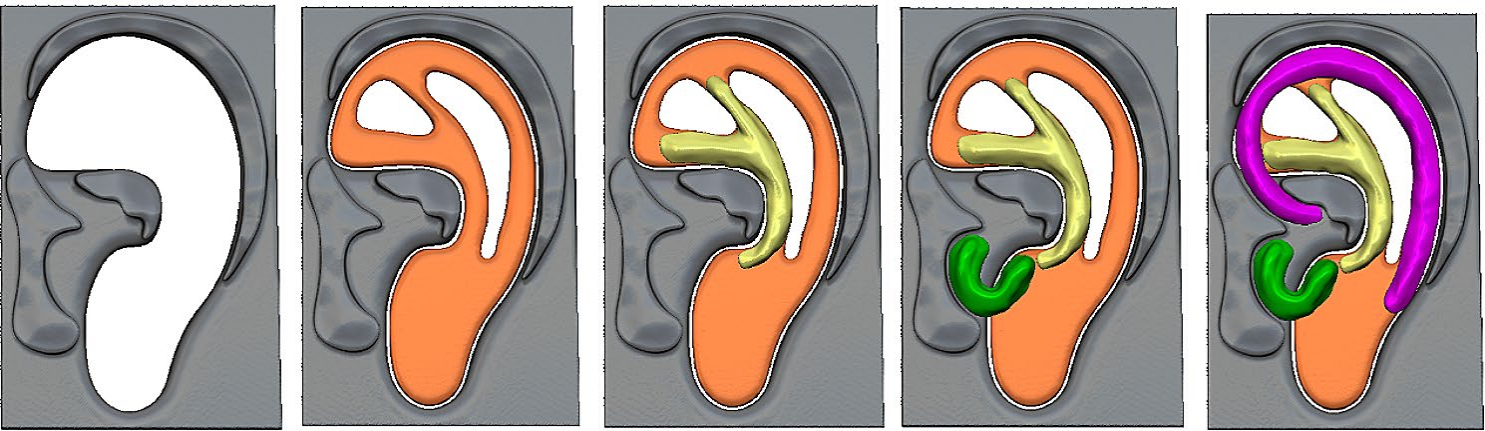
Virtual sequence of the cartilage pieces assembly to shape the ear framework over the 3D mold. The helix is represented in pink, the antihelix in yellow, the tragus-antitragus complex is displayed in green, and the base is orange

Furthermore, we used *Geomagic Freeform Plus* software to design several templates of the helix, antihelix, tragus-antitragus and a base that supports the entire assembly relying on anatomical landmarks of the specular virtual ear. Moreover, we developed a mold to facilitate the creation of the cartilage framework of the new ear (Fig. 2). All these components served as guiding tools for precise cartilage cutting and carving during the surgical intervention.

### 3D printing and sterilization

After finalizing the designs and obtaining consensus among the authors, all components were produced through additive manufacturing techniques. Initially, a set of templates was 3D printed using Formlabs Grey resin. These templates allowed surgeons to practice ear reconstruction on expanded polystyrene sheets before the actual operation. Additionally, a comprehensive pack, including the dental splint, was manufactured using biocompatible BioMed Clear V1 resin suitable for clinical use in the operating room. This resin possesses USP class IV certification, ensuring its suitability for prolonged contact with patient organs [30]. Both sets of components were fabricated using the stereolithography technique with a Formlabs Form 2 3D printer (Formlabs Inc., Somerville, MA, USA).

As part of the preliminary assessment in the simulation scenario, we further utilized 3D printing technology to create the skull phantom and an additional dental splint. Both items were fabricated using PLA material employing the fused deposition modeling (FDM) technique on an Ender 3 3D printer. Furthermore, a double extruder Ultimaker 3 Extended desktop 3D printer was employed to produce the AR reference marker for the AR application [18], also using PLA material. The decision to use a different 3D printer to create the AR reference marker was driven by the need to print it with two colors. This required a double extruder, which is found in Ultimaker 3 Extended but not in Ender 3.

Before their use during surgery, all components were subjected to the necessary sterilization procedures in compliance with the operating room’s sterilization requirements. The resin components underwent sterilization using ethylene oxide (EtO) at 55 °C, while the PLA components were sterilized at 37 °C [24, 31]. This lower temperature was chosen specifically to prevent PLA deformation [32].

### Augmented reality application

**Fig. 3:**
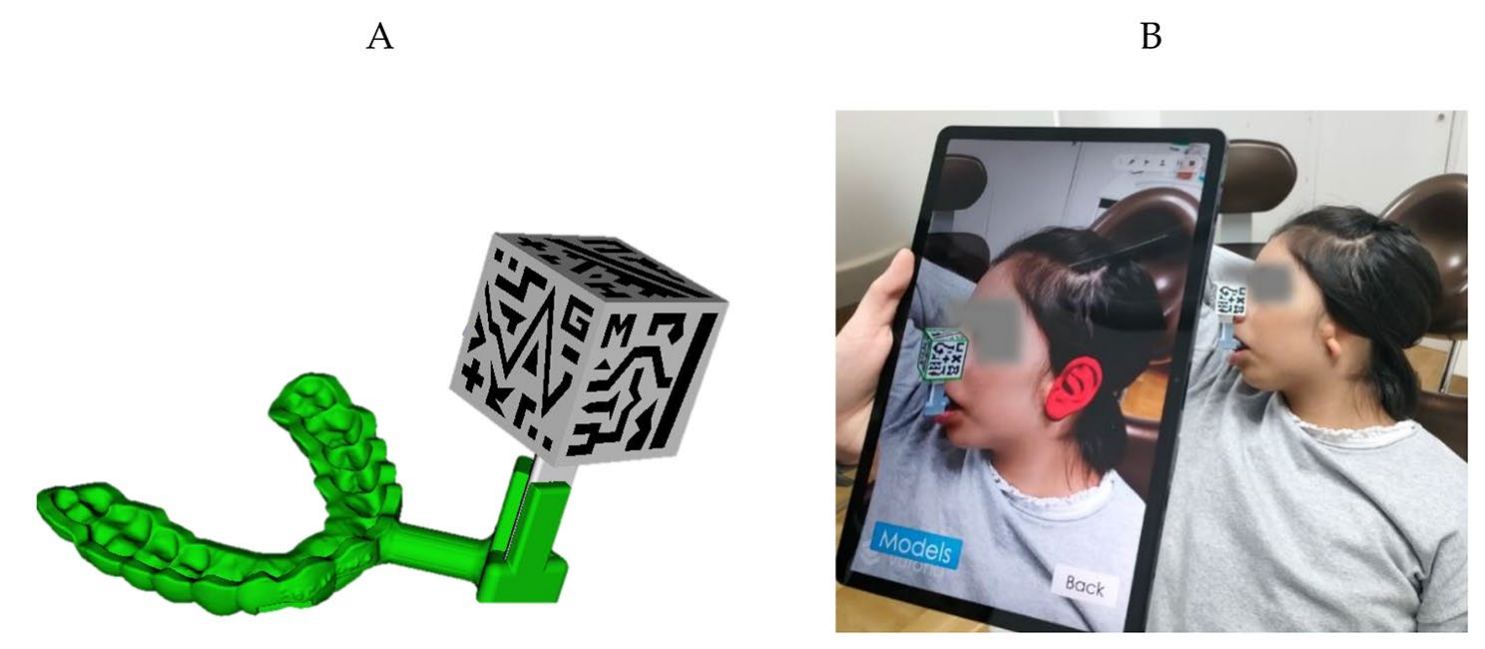
(**A**) Virtual design of the dental splint with adaptor holding the AR reference marker. (**B**) AR application test prior to surgery to guarantee adequate projection of the specular ear, in red

We created an AR application for tablets that served as a guide for accurately placing the new ear on the patient’s head during the surgical procedure. We utilized a Samsung Galaxy Tab S7 Tablet during the whole experience, although the application is compatible with any Android device. The AR application was developed on Unity 2019.3.0f6 using C# programming language. It is based on a previously proposed AR application described in [24]. The custom splint designed in this study incorporates a small support to hold the AR reference marker. Its cubic shape contains specific patterns on each face that are easily identifiable by the AR application (Fig. 3A).

The AR reference marker was detected within the camera’s FOV of the tablet thanks to the Vuforia software development kit (SDK) (Parametric Technology Corporation Inc., Boston, MA, USA). According to the identified AR reference marker pose, the application renders a virtual ear in its designated position, a virtual representation of the dental splint and the patient’s teeth (SuppVideo2_RehearsalBeforeSurgery). Proper alignment between the virtual and real worlds can be visually verified by checking the AR reference marker silhouette overlaid onto the physical marker (Fig. 3B). The application incorporates a color-coded frame that transitions from green to red, indicating when tracking is lost during the procedure. Additionally, the application includes interactive elements such as buttons and slide bars associated with each virtual model. These controls empower surgeons to personalize the display by adjusting the visibility and transparency of the virtual models according to their preferences.

### Evaluation of the system

**Fig. 4:**
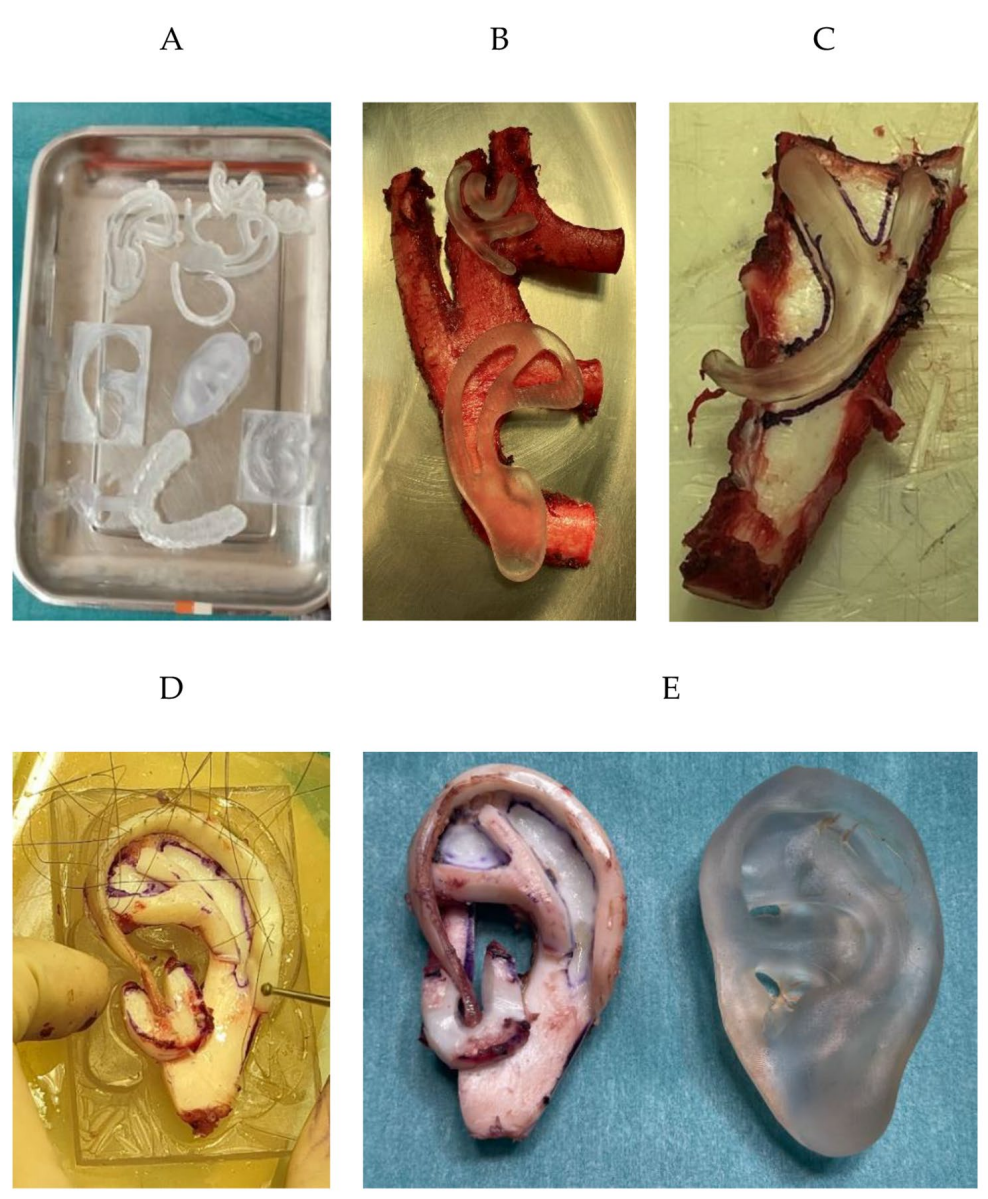
Reconstruction of the cartilage framework based on the 3D printed templates and molds. (**A**) 3D printed patient-specific components ready in the operating room. (**B**) Fitting of the 3D templates on the extracted rib cartilage. (**C**) Antihelix piece outlined on cartilage based on 3D templates. (**D**) Cartilage framework assembled with the help of the mold. (**E**) Final construction next to a 3D printed template of the specular ear

We initially evaluated our system in a simulation scenario that included the skull phantom, the PLA dental splint and the AR reference marker. The evaluation methodology and the corresponding results of these experiments are presented in SuppDocument1. After determining the accuracy of our solution in a controlled environment, we tested it in the actual surgery. SuppVideo3_SurgicalIntervention offers a summary of the surgical steps followed during the intervention. It began by extracting costal cartilage from the 5th to the 8th ribs. The sterile patient-specific 3D printed templates were used to harvest the four pieces of the new ear (Fig. 4A). Specifically, they guided the delineation of the edge of each ear component over the cartilage, replacing the traditional X-ray film (Fig. 4B and Fig. 4C). The cartilage framework’s base was formed using cartilage obtained from the 6th and 7th ribs. To construct the antihelix and tragus-antitragus pieces, the 5th rib and the remaining cartilage from the 6th rib were employed. Furthermore, the helix was crafted using cartilage sourced from the 8th rib. Any remaining unused cartilage was carefully placed in a subcutaneous pocket before fully closing the wound. This strategic approach was taken in preparation for the second stage of the procedure, which will focus on reconstructing the concha.

After creating all components, the ear was mounted on the 3D printed mold, subsequently adding the base, antihelix, tragus-antitragus complex, and helix (Fig. 4D). All pieces were assembled using 5/0 Steelex® stainless steel threads, double-armed with straight GS atraumatic needles (Ear Set, B. Braun, Melsungen, Germany). The final cartilage reconstruction next to the 3D printed specular ear template is shown in Fig. 4E.

The cartilaginous scaffold was subsequently introduced on a skin pocket carved within the patient’s head. The cutaneous approach to create this pocket is the most important part of the surgery, as the final surgical outcome depends on this stage. During the surgical procedure, the malformed cartilage was extracted and gently detached from the adjacent skin. This step must be performed carefully to avoid perforation and damage to the subdermal plane. Two size 10 Blake drains were then introduced in the pocket to adapt the skin of the cutaneous pocket to the entire topography of the cartilaginous framework.

**Fig. 5:**
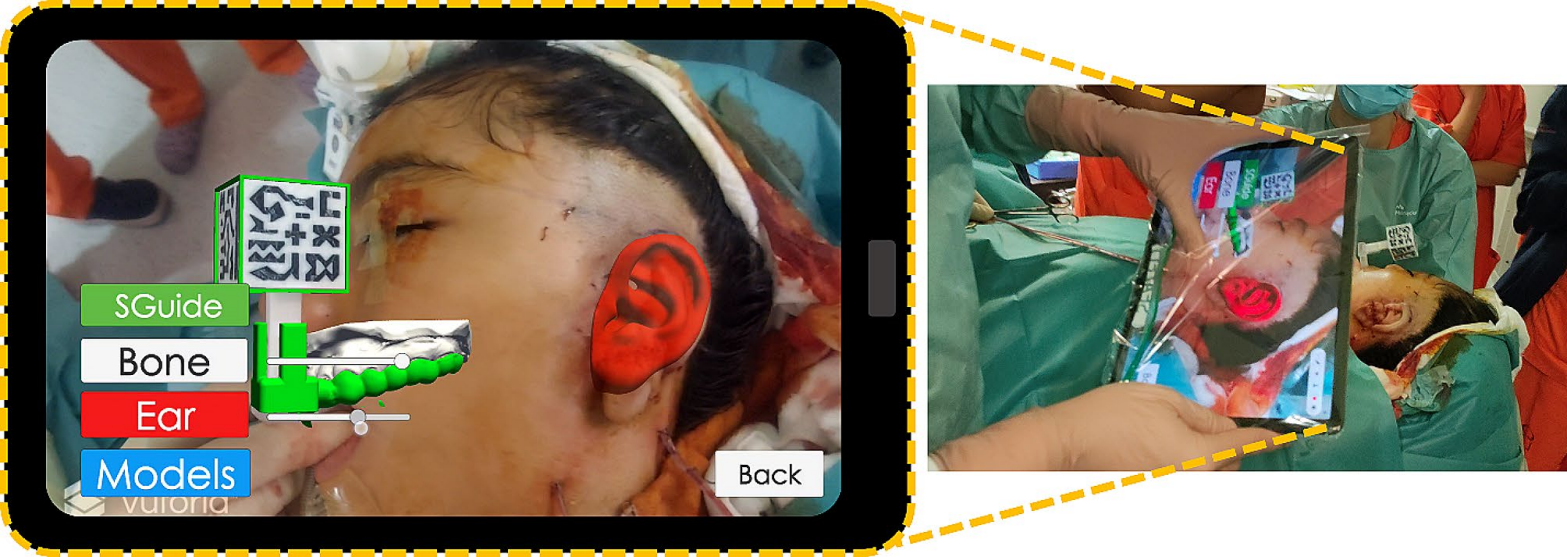
AR application during the surgical procedure. The virtual plan of the specular ear is displayed in red to guide the placement of the cartilaginous framework

To determine the appropriate position and orientation of the scaffold in the patient’s head, the surgeons relied on the information provided by the AR application. First, they adjusted the dental splint onto the patient’s upper teeth and attached the AR reference marker. Next, they inserted the tablet into a sterile eShield™ cover, designed for use during surgical procedures [33]. The surgeons initialized the AR application and directed the tablet camera towards the AR reference marker to see the precise location of the ideal ear (Fig. 5). They gently adjusted the position and rotation of the cartilaginous framework within the pocket according to the visualization of the virtual ear. Once finished, alignment was verified with traditional reference measurements. To conclude, the surgeons used the Blake drains to create a vacuum and closed the wound.

After the procedure, intraoperative 3D photographs of the head and both ears were acquired using an Artec EVA ® (Artec3D, Senningerberg, Luxembourg) structured light scanner. This information allowed a comprehensive comparison between pre-operative virtual plan and a post-procedure patient´s head virtual 3D model (SuppVideo4_PostoperativeScanVSIdealPlan). The focus of the evaluation was the precision of the reconstructed ear shape as well as its final positioning. Specifically, we compared the STL file of the virtual plan, including facial soft tissue and the specular 3D virtual ear in its ideal planned position, with the patient’s geometry scanned postoperatively. Both 3D models were aligned in 3D Slicer with a focus on their most similar surfaces (i.e. eyes, forehead and nose).

SuppContent_3DModels contains all 3D models employed for performing the analyses presented in this work. This includes the original 3D model of the patient’s head retrieved from the head CT scan, the ideal plan derived from this model (including the specular ear), and the postoperative result. Furthermore, SuppContent_3DModels contains the ideal phantom reference for the simulation scenario, as well as all ear crops from each of the user’s experiments.

## Results

To initially evaluate the accuracy of our technology, we performed some experiments in a controlled scenario. Overall, we obtained a mean Euclidean distance of 2.2 ± 1.7 mm, considering the absolute value of data measured from all experiments (SuppDocument1).

**Fig. 6:**
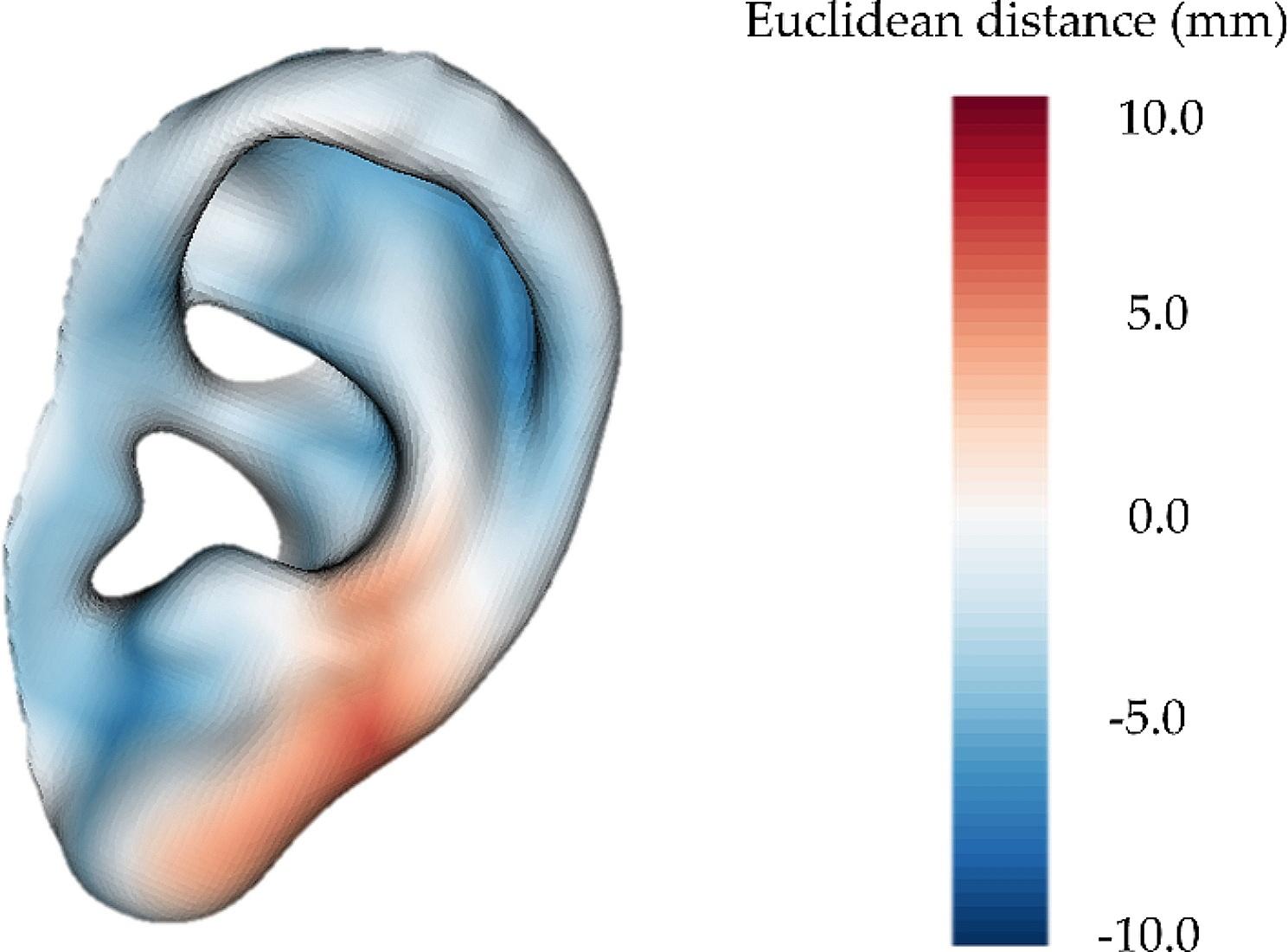
Distance map between the ear reconstructed at surgery and the ideal plan

The evaluation of the surgical outcomes began by analyzing ear reconstruction from 3D printed templates. A morphometric analysis comparing the shapes of the surgical outcome and the ideal plan reveals a mean Euclidean distance between models of 2.2 ± 1.3 mm, considering absolute values. A distance map reflecting these differences is presented in Fig. 6. A maximum distance of 4.5 mm was measured in the posterior region of the auricle. Considering length as the longest dimension of the ear and width as the shortest metric in the perpendicular direction, the differences in length and width between the final ear and the specular one are 3.1 mm and 1.3 mm, respectively.

**Fig. 7:**
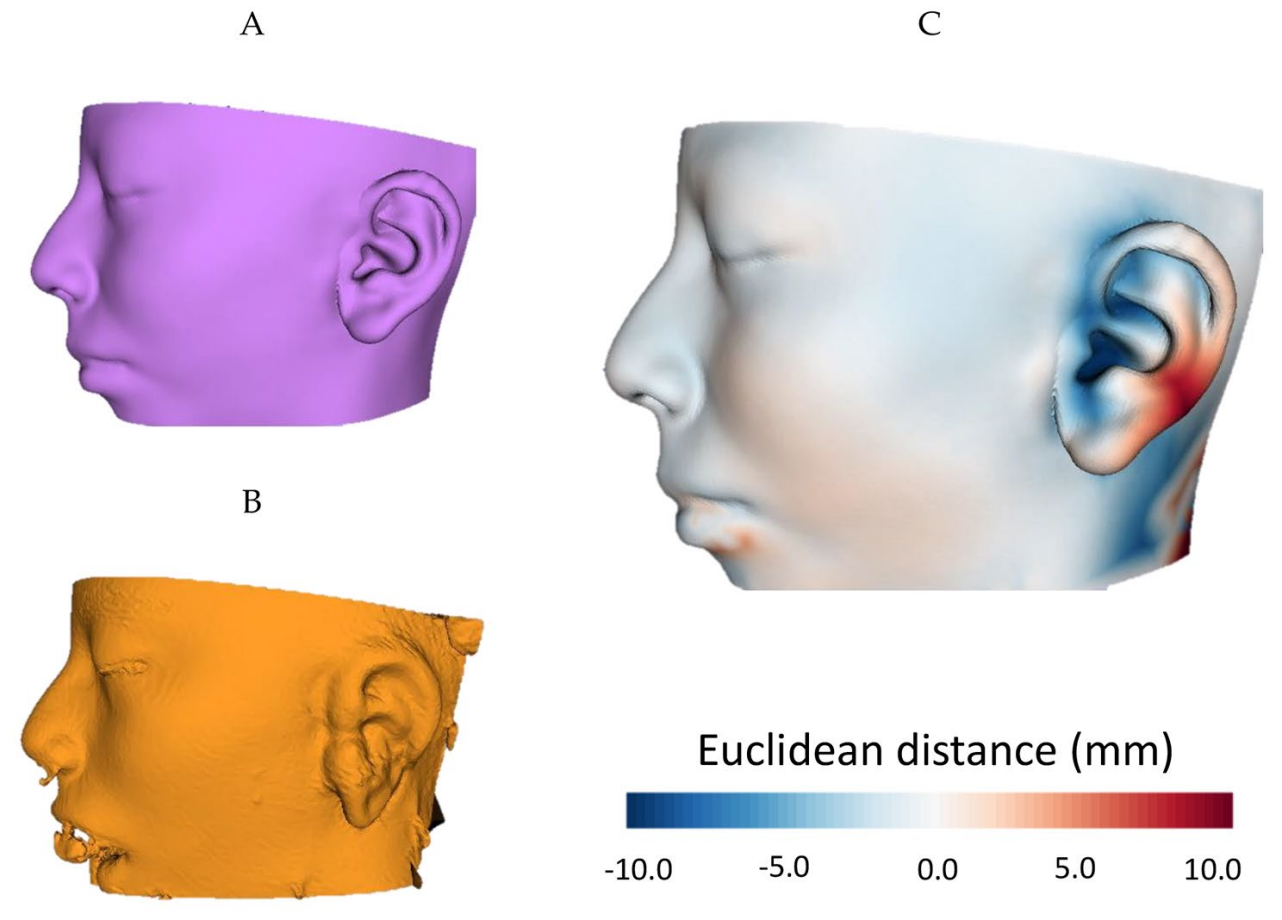
(**A**) Ideal plan with perfect ear reconstruction; (**B**) 3D model representing surgical outcomes obtained from intraoperative surface scan; (**C**) Euclidean distance map comparison between 3D models in (**A**) and (**B**)

To analyze ear placement in the surgical scenario, Fig. 7A represents the preoperative ideal plan, showcasing a perfectly reconstructed and positioned ear. Besides, Fig. 7B presents a 3D model of the patient’s head obtained from the post-operative surface scan. A distance map comparing the Euclidean distance between both models is depicted in Fig. 7C. It provides a visual representation of the differences between the preoperative ideal plan and the post-operative outcome. The reference model used to define positive or negative distances is the ideal plan with perfect ear positioning.

**Fig. 8:**
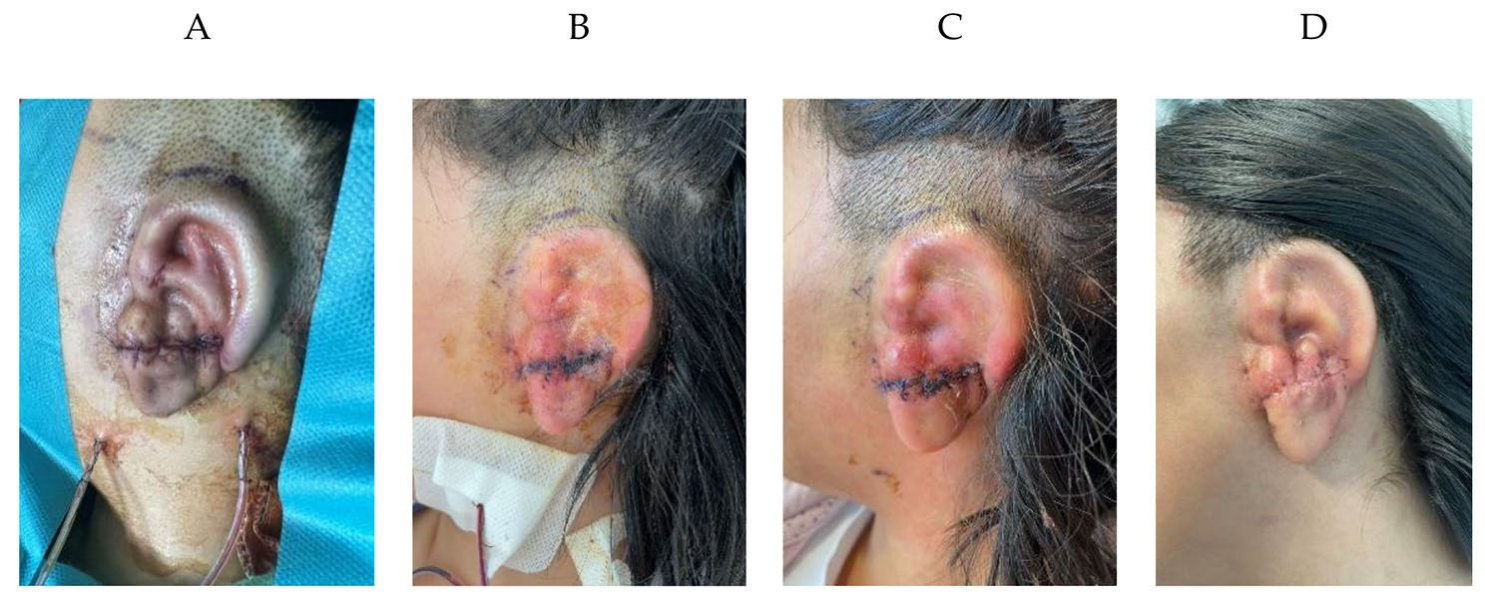
Patient follow-up. (**A**) Intraoperative result. (**B**) 3 days postoperative. (**C**) 8 days postoperative. (**D**) 20 days postoperative

Following registration, the mean Euclidean distance between the models was 2.7 ± 2.4 mm. This value encompasses all errors resulting from variations in ear shape and positioning. Notably, variations were observed in specific facial regions, such as the cheeks, with a maximum deviation of 1.3 mm; and the inferior lip, presenting maximum deviation of 4.8 mm. These divergences can be attributed to facial gestures resulting from intubation during the procedure. Regarding the target ear, a minimum deviation of -10.2 mm is obtained on its anterior section, whereas a maximum distance of 9.7 mm is measured in the helix region. This means that the ear was slightly rotated around the axial axis. Little to no displacement can be observed in any other rotation or translation axes. Fig. 8 showcases the follow up of the patient during the first 20 days after the surgical procedure.

## Discussion

Up to date, correction of microtia has been a highly subjective procedure that strongly depended on the surgeons’ dexterity and artistic abilities. In this work, we have explored the combination of 3D printing with AR on a hand-held device to improve and objectify these surgical procedures. Based on the work presented in [24], our AR application was integrated with the design and manufacturing of patient-specific 3D templates based on medical images. These advancements played a crucial role throughout the entire surgical procedure, facilitating the creation and placement of a reconstructed ear on the patient’s head.

Our solution was evaluated in a controlled environment and during an actual surgery. In the first case, we demonstrated the accuracy of the system, obtaining a mean Euclidean distance of 2.2 ± 1.7 mm, which is comparable to the state-of-the-art in AR projection accuracy [24, 34–36]. Then, we implemented our system in an actual surgery, where we measured the effectiveness of the 3D printed templates in the operating room (OR) by analyzing the disparities between the final and the ideal ears. The reconstructed ear demonstrates an average deformation of 2.2 ± 1.3 mm compared to the ideal one. The analysis reveals no discernible pattern in the spatial distribution of the error that could be attributed to defects in the 3D printed templates. Consequently, we are confident that the results obtained are appropriately associated with the matter under study.

While numerous studies have aimed to enhance objectivity and reproducibility in auricle reconstruction, only a few have provided precise accuracy metrics supporting their claims. One of them is published by Zhou et al. in [37]. Like us, they utilized 3D patient-specific templates to guide ear reconstruction during the surgical intervention of 40 patients. Additionally, they employed traditional X-Ray templates in the same cases and analyzed the outcomes achieved from both techniques. When comparing the accuracy of the X-ray film and 3D printed templates, they reported average length errors of 1.8 ± 1.4 mm and 0.4 ± 0.4 mm, respectively. For average width errors, they obtained 1.3 ± 0.9 mm for X-ray templates and 0.3 ± 0.5 mm for 3D printed templates, relative to the contralateral healthy ear. In our case, the differences in length and width were 3.1 mm and 1.3 mm, respectively. Note that the surgical ear was scanned and measured right after surgery when it presented a high inflammatory response. Consequently, the ear was enlarged and slightly deformed relative to the expected future shape. This enlargement is reflected in the prevalence of blue color in Fig. 6.

The primary distinction between our 3D printing solution and [37] is that they only create one 3D template per user, incorporating all ear components (base, helix, antihelix, and tragus-antitragus complex) in a single structure. In our case, we 3D printed all elements separately to use them as guidance for cartilage cutting, enhancing the precision and customization of the reconstruction process.

After creating the cartilage framework, it must be inserted within the patient’s head. Nuri et al. also explored AR as a guiding tool in this step [38]. They developed an AR application for Microsoft HoloLens, which projected an image of the expected outcome directly onto the patient. They reported an alignment error of 2 mm when compared to the original transparent film technique. However, their method relied on manual alignment between the virtual and real worlds through three reference landmarks on the patient’s face. This approach is generally not recommended as it can introduce significant errors and lead to high variability between repetitions [39].

In contrast, our approach employs a patient’s specific guide and an AR reference marker to achieve automatic registration and minimize human error. By incorporating these elements, we aim to enhance the accuracy and consistency of the alignment process. Furthermore, our solution is implemented on a tablet application, whereas their approach utilizes a head-mounted display. In our case, we intended to offer a shared visualization during guidance, rather than confining the experience solely to the user wearing the glasses. By adopting a tablet-based implementation, we aimed to facilitate cooperative decision-making, enabling multiple surgeons to participate in the process.

Overall, the reconstructed ear of our work has a positioning error of 2.7 ± 2.4 mm with respect to the surgical plan, presenting higher deviation in the axial axis of rotation. It is worth noting that the ideal plan displays a greater projection of the ear. During this surgery, the retroauricular groove was intentionally left unreconstructed, resulting in the entire ear being flush with the surface of the head and presenting a flattened disposition. This issue could justify the increase of the Euclidean distance in the analysis, but it will be addressed and rectified in the subsequent second-stage surgery. Apart from this, the overall alignment of the new ear with respect to the desired plan demonstrates remarkable accuracy.

Previous studies have sought to establish baseline differences in contralateral ears among the general population, revealing that individuals with two fully developed ears often exhibit a certain degree of asymmetry [40]. For example, in a cross-sectional observational study in [41], the authors analyze left and right ear symmetry in 505 male and female participants of different age groups. Across the entire cohort, average differences of 0.5 mm and 0.2 mm were observed between contralateral ears in length and width, respectively. However, within the same individual, these differences can reach 1.7 mm in height and 4.0 mm in width [42]. These aspects were not considered in our planning step, where the objective was a fully symmetrical ear, but could be an area to explore to obtain a more natural result.

One of the major benefits of the proposed workflow is its affordability and rapid implementation. The entire process, from initial diagnosis and treatment indication to surgery, was completed in under three weeks, including the development of the AR application and the design and 3D printing of surgical guides. The financial and temporal aspects of the 3D printing process will differ depending on whether it is done in-hospital, leveraging point-of-care manufacturing [43], or with external providers. In our case, opting for the latter, we managed to keep the total cost of 3D printing cost below 1000€. Regarding the AR application, we followed similar steps to those presented in our previous work [18], for which instructions and code are available. However, it was possible to quickly adapt a new AR application to this procedure thanks to our previous experience in AR, Unity, and 3D Slicer. The developed software is not approved as a medical device and was evaluated in the context of a pilot research protocol. In the European Union, both medical 3D printing and software must adhere to regulation (EU) 2017/745 of the European Parliament and of the Council of 5 April 2017 on medical devices. While it’s challenging to assess the costs associated with equivalent commercial AR applications, the streamlined nature of our workflow, its rapid execution, and minimal hardware demands significantly bolster the potential of this technology in the treatment of microtia.

## Conclusions

The ultimate goal in microtia ear reconstruction is the achievement of a perfect symmetry with the contralateral healthy auricle. Whilst the fabrication of a framework determines the fine structure, other critical surgical decisions, such as the cranioauricular angle and the size and tridimensional localization of the reconstructed ear, determine the overall postoperative outcome. Surgeons commonly use planimetric marker lines when crafting the cartilage framework and guiding the final positioning [44].

In this work, we have demonstrated the potential of combining AR and patient-specific 3D templates to enhance the precision and efficacy of these procedures. Specifically, the 3D printed patient-specific templates improve objectivity while enabling surgeons to practice the surgery beforehand, gaining confidence in their approach. Furthermore, the AR application provides natural guidance for ear positioning, aiding surgeons in real-time corrections without the need for constant anatomical measurements. Despite the individual implementation of these 3D technologies having been previously explored for the correction of microtia, to our knowledge, this is the first work presenting their combined use. Our proposal leverages the advantages of both technologies, achieving promising results.

This work has been possible thanks to the multidisciplinary collaboration between surgeons and engineers, which facilitates the invention of novel surgical techniques to treat patients with complex challenging reconstructions. We hope we have laid the foundation for a more objective, precise, and collaborative approach to microtia correction. These advancements hold promise for improving surgical outcomes, reducing errors, and enhancing patient satisfaction.

## Data Availability

The data supporting the findings of this study has been deposited in Zenodo at https://zenodo.org/records/10958624.

## Abbreviations

3D: Three-dimensional
AR: Augmented reality
FDM: Fused deposition modelling
OR: Operating room
PLA: Polylactic acid
SDK: Software development kit

## References

[1] Carl B, Bopp M, Saß B, Pojskic M, Nimsky C (2019). Augmented reality in intradural spinal tumor surgery. Acta Neurochir.

[2] Farshad M, Fürnstahl P, Spirig JM (2021). First in man in-situ augmented reality pedicle screw navigation. North Am Spine Soc J (NASSJ).

[3] Shuhaiber JH (2004). Augmented reality in surgery. Arch Surg.

[4] Luzon JA, Stimec BV, Bakka AO, Edwin B, Ignjatovic D (2020). Value of the surgeon’s sightline on hologram registration and targeting in mixed reality. Int J Comput Assist Radiol Surg.

[5] Balani MS, Tümler J, Chen JYC, Fragomeni G (2021). Usability and user experience of interactions on VR-PC, HoloLens 2, VR Cardboard and AR Smartphone in a Biomedical Application. Virtual, augmented and mixed reality.

[6] Ivan ME, Eichberg DG, Di L, Shah AH, Luther EM, Lu VM (2021). Augmented reality head-mounted display–based incision planning in cranial neurosurgery: a prospective pilot study. NeuroSurg Focus.

[7] Deng W, Li F, Wang M, Song Z (2013). Easy-to-use augmented reality neuronavigation using a Wireless Tablet PC. Stereotact Funct Neurosurg.

[8] Contreras López WO, Navarro PA, Crispin S (2019). Intraoperative clinical application of augmented reality in neurosurgery: a systematic review. Clin Neurol Neurosurg.

[9] Inoue D, Cho B, Mori M, Kikkawa Y, Amano T, Nakamizo A (2013). Preliminary study on the clinical application of augmented reality neuronavigation. Journal of neurological surgery part A. Cent Eur Neurosurg.

[10] Joda T, Gallucci GO, Wismeijer D, Zitzmann NU (2019). Augmented and virtual reality in dental medicine: a systematic review. Comput Biol Med.

[11] Ackermann J, Liebmann F, Hoch A, Snedeker JG, Farshad M, Rahm S (2021). Augmented reality based Surgical Navigation of Complex Pelvic Osteotomies—A feasibility study on cadavers. Appl Sci.

[12] Elmi-Terander A, Burströ G, Nachabe R, Skulason H, Pedersen K, Fagerlund M et al. Pedicle Screw Placement Using Augmented Reality Surgical Navigation With Intraoperative 3D Imaging A First In-Human Prospective Cohort Study. www.spinejournal.com. 10.1097/BRS.0000000000002876 PMC6426349 30234816

[13] Zhou C, Zhu M, Shi Y, Lin L, Chai G, Zhang Y (2017). Robot-assisted surgery for Mandibular Angle Split Osteotomy using augmented reality: preliminary results on Clinical Animal Experiment. Aesthetic Plast Surg.

[14] Stuart J, Baxter H, Khan AR, Peters T, Abhari K, Baxter JSH et al. Training for Planning Tumour Resection: Augmented Reality and Human Factors Article in IEEE transactions on bio-medical engineering · Training for Planning Tumour Resection: Augmented Reality and Human Factors. IEEE TRANSACTIONS ON BIOMEDICAL ENGINEERING [Internet]. 2015;62(6). https://www.researchgate.net/publication/270220608. 10.1109/TBME.2014.2385874 25546854

[15] Abdel Al S, Chaar MKA, Mustafa A, Al-Hussaini M, Barakat F, Asha W (2020). Innovative Surgical Planning in Resecting Soft tissue sarcoma of the Foot using augmented reality with a smartphone. J Foot Ankle Surg.

[16] Heinrich F, Joeres F, Lawonn K, Hansen C (2019). Comparison of Projective Augmented reality concepts to Support Medical needle insertion. IEEE Trans Vis Comput Graph.

[17] García-Sevilla M, Moreta‐Martinez R, García‐Mato D, Pose‐Díez‐de‐la‐Lastra A, Pérez‐Mañanes R, Calvo‐Haro JA et al. Augmented reality as a tool to guide psi placement in pelvic tumor resections. Sensors. 2021;21(23). 10.3390/s21237824 PMC8659846 34883825

[18] Moreta-Martinez R, García-Mato D, García-Sevilla M, Pérez-Mañanes R, Calvo-Haro JA, Pascau J. Combining augmented reality and 3d printing to display patient models on a smartphone. Journal of Visualized Experiments [Internet]. 2019;2020(155). https://www.jove.com/video/60618/combining-augmented-reality-3d-printing-to-display-patient-models-on. 10.3791/60618 31957749

[19] Hatz CR, Msallem B, Aghlmandi S, Brantner P, Thieringer FM (2020). Can an entry-level 3D printer create high-quality anatomical models? Accuracy assessment of mandibular models printed by a desktop 3D printer and a professional device. Int J Oral Maxillofac Surg.

[20] Jiang M, Chen G, Coles-Black J, Chuen J, Hardidge A (2020). Three-dimensional printing in orthopaedic preoperative planning improves intraoperative metrics: a systematic review. ANZ J Surg.

[21] Punyaratabandhu T, Liacouras PC, Pairojboriboon S. Using 3D models in orthopedic oncology: presenting personalized advantages in surgical planning and intraoperative outcomes. 3D Printing. in Medicine. 2018;4(1). 10.1186/s41205-018-0035-6 PMC6261090 30649645

[22] De La Peña A, De La Peña-Brambila J, Pérez-De La Torre J, Ochoa M, Gallardo GJ. Low-cost customized cranioplasty using a 3D digital printing model: a case report. 3D Printing. in Medicine. 2018;4(1). 10.1186/s41205-018-0026-7 PMC5954791 29782609

[23] Samaila EM, Negri S, Zardini A, Bizzotto N, Maluta T, Rossignoli C et al. Value of three-dimensional printing of fractures in orthopaedic trauma surgery. J Int Med Res. 2019;48(1). 10.1177/0300060519887299 PMC7262838 31813322

[24] Moreta-Martinez R, Pose-Díez-de-la-Lastra A, Calvo-Haro JA, Mediavilla-Santos L, Pérez-Mañanes R, Pascau J. Combining Augmented Reality and 3D Printing to Improve Surgical Workflows in Orthopedic Oncology: Smartphone Application and Clinical Evaluation. Sensors 2021, Vol 21, Page 1370. 2021;21(4):1370–1370. 10.3390/s21041370 PMC7919470 33672053

[25] Luquetti DV, Heike CL, Hing AV, Cunningham ML, Cox TC (2012). Microtia: epidemiology and genetics. Am J Med Genet A.

[26] Baluch N, Nagata S, Park C, Wilkes GH, Reinisch J, Kasrai L (2014). Auricular reconstruction for microtia: a review of available methods. Plast Surg (Oakv).

[27] Firmin F (2010). State-of-the-art autogenous ear reconstruction in cases of microtia. Adv Otorhinolaryngol.

[28] Firmin F, Marchac A (2011). A novel algorithm for autologous ear Reconstruction. Semin Plast Surg.

[29] Yotsuyanagi T, Yamashita K, Yamauchi M, Nakagawa T, Sugai A, Kato S (2019). Establishment of a standardized technique for Concha-type Microtia-How to incorporate the cartilage frame into the remnant ear. Plast Reconstr Surg Glob Open.

[30] United States Pharmacopeial Convention. The United States Pharmacopeia [Internet], Rockville MD. USA, 2012; Volume 1. Available online: https://www.usp.org/ (accessed on Feb 10, 2022); 94 p. https://www.usp.org/.

[31] Zislis T, Martin SA, Cerbas E, Heath JR, Mansfield JL, Hollinger JO (1989). A scanning electron microscopic study of in vitro toxicity of ethylene-oxide-sterilized bone repair materials. J Oral Implantol.

[32] Sharma N, Cao S, Msallem B, Kunz C, Brantner P, Honigmann P (2020). Effects of Steam sterilization on 3D printed Biocompatible Resin materials for Surgical Guides-An Accuracy Assessment Study. J Clin Med.

[33] eShield Covers -. Bring Smart Devices into Sterile Surgical Environments [Internet]. [cited 2023 May 3]. https://www.whitneymedicalsolutions.com/eshield-covers.

[34] Solbiati M, Passera KM, Rotilio A, Oliva F, Marre I, Goldberg SN (2018). Augmented reality for interventional oncology: proof-of-concept study of a novel high-end guidance system platform. Eur Radiol Experimental.

[35] Wen R, Chng CB, Chui CK. Augmented reality guidance with multimodality imaging data and depth-perceived interaction for robot-assisted surgery. Robotics. 2017;6(2).

[36] Carl B, Bopp M, Voellger B, Saß B, Nimsky C (2019). Augmented reality in transsphenoidal surgery. World Neurosurg.

[37] Zhou J, Pan B, Yang Q, Zhao Y, He L, Lin L (2016). Three-dimensional autologous cartilage framework fabrication assisted by new additive manufactured ear-shaped templates for microtia reconstruction. J Plast Reconstr Aesthet Surg.

[38] Nuri T, Mitsuno D, Otsuki Y, Ueda K (2020). Augmented reality technology for the positioning of the auricle in the Treatment of Microtia. Plast Reconstr Surg Glob Open.

[39] Mitsuno D, Ueda K, Hirota Y, Ogino M (2019). Effective application of mixed reality device HoloLens: simple manual alignment of Surgical Field and Holograms. Plast Reconstr Surg.

[40] Claes P, Reijniers J, Shriver MD, Snyders J, Suetens P, Nielandt J (2015). An investigation of matching symmetry in the human pinnae with possible implications for 3D ear recognition and sound localization. J Anat.

[41] Japatti SR, Engineer PJ, Reddy BM, Tiwari AU, Siddegowda CY, Hammannavar RB (2018). Anthropometric Assessment of the normal adult human ear. Ann Maxillofac Surg.

[42] Abaza A, Ross A. Towards understanding the symmetry of human ears: A biometric perspective. In: 2010 Fourth IEEE International Conference on Biometrics: Theory, Applications and Systems (BTAS) [Internet]. Washington, DC, USA: IEEE; 2010 [cited 2024 Mar 21]. pp. 1–7. http://ieeexplore.ieee.org/document/5634535/.

[43] Calvo-Haro JA, Pascau J, Asencio-Pascual JM, Calvo-Manuel F, Cancho-Gil MJ et al. Del Cañizo López JF,. Point-of-care manufacturing: a single university hospital’s initial experience. 3D Printing in Medicine. 2021;7(1):11. 10.1186/s41205-021-00101-z PMC8061881 33890198

[44] Gu Q, He L, Zheng B (2021). A feasibility study of real time dynamic three-dimensional auricular image guidance based on augmented reality. J Plast Reconstr Aesthet Surg.

